# Post-procedure sedation and apnea linked to ion channel variant: a case report on dexmedetomidine-propofol interaction

**DOI:** 10.3389/fphar.2025.1549540

**Published:** 2025-04-25

**Authors:** Gabriele Stocco, Anna Galletti, Marianna Lucafò, Nagua Giurici, Debora Curci, Sara Solidoro, Valentina Kiren, Anna Monica Bianco, Egidio Barbi, Pio D’Adamo

**Affiliations:** ^1^ Clinical Department of Medical, Surgical and Health Sciences, University of Trieste, Trieste, Italy; ^2^ Advanced Translational Diagnostic Laboratory, Institute for Maternal and Child Health-IRCCS Burlo Garofolo, Trieste, Italy; ^3^ Pediatric Department, Institute for Maternal and Child Health-IRCCS Burlo Garofolo, Trieste, Italy; ^4^ Department of Life Sciences, University of Trieste, Trieste, Italy; ^5^ Department of Pediatric Oncohematology Institute for Maternal and Child Health-IRCCS Burlo Garofolo, Trieste, Italy; ^6^ Institute for Maternal and Child Health-IRCCS Burlo Garofolo, Trieste, Italy; ^7^ Genetics Department, Institute for Maternal and Child Health-IRCCS Burlo Garofolo, Trieste, Italy

**Keywords:** post awakening apnea, re-sedation, dexmedetomidine, propofol, *SCN9A* gene variant

## Abstract

We report a case of post-awakening recurrent episodes of spontaneous re-sedation and apnea with severe desaturation after procedural sedation with dexmedetomidine and propofol in a leukemic adolescent with an ionic channel variant. The mutation is located in the 3′-UTR regulatory region of SCN9A. We speculate that this variant may affect the stability of the mRNA, making the patient more susceptible to the combined effects of propofol and dexmedetomidine. This is the first pediatric report of late onset re-sedation with apnoea after combined sedation with propofol and dexmedetomidine highlighting the risk of adverse events in selected patients with a genetic increased susceptibility. If validated by further studies, pharmacogenetic testing may be implemented to provide personalized therapies in patients needing anesthesia.

## Introduction

Due to the availability of short-acting sedatives like propofol and those minimally impacting respiratory drive such as dexmedetomidine, combined with high standards of training and monitoring, sedation and analgesia have become routine with excellent safety profiles. However, rare adverse events, particularly in individuals with unique pharmacogenetic profiles, remain a critical area of concern. This report aims to increase physician awareness of delayed sedation and apnea in pediatric patients, emphasizing the potential role of pharmacogenetics. Ion channel variants can influence patient responses to medications: for example, variants in the sodium voltage-gated channel alpha subunit 9 (SCN9A) gene, may affect response to propofol (Zhong et al., 2017).

## Case description

A 14-year-old girl with lymphatic leukemia was admitted for a scheduled lumbar puncture with methotrexate spinal infusion and initiation of the second block of cytarabine as per the AIEOP LLA 2017 protocol. Her ongoing medications included thioguanine, acyclovir, amlodipine, lansoprazole, and leuprorelin.

Following fasting recommendations, she had consumed clear fluids 3 h before the procedure.

At admission, her physical evaluation was unremarkable: SpO2 99%, heart rate 85 bpm, and blood pressure 106/70 mmHg. Her weight was 55.4 kg.

Due to emerging evidence linking cumulative propofol doses to neurological damage, the team adopted a propofol-sparing protocol (Banerjee et al., 2019). Therefore, in the last months, we adopted a new propofol-sparing protocol in our ward based on 1 mcg/kg in 10 min of dexmedetomidine infusion premedication before propofol boluses.

The patient’s sedation was initiated with a bolus of 1 mcg/kg of dexmedetomidine infused over 10 min. After stopping the dexmedetomidine bolus, we slowly infused a first dose of 30 mg of propofol over 2 min.

The patient experienced regurgitation of a minimal quantity of gastric fluids without desaturation or abnormalities in her breathing pattern. The procedure was continued because the regurgitation appeared minimal, and the breathing pattern was stable. We kept a mask close, delivering 90% oxygen to the patient’s mouth. A bolus of 8 mg of ondansetron was administrated, followed by additional boluses of propofol (total dose 290 mg, 5.2 mg/kg) to achieve moderate to deep sedation for a comfortable procedure. The puncture presented some technical difficulties, requiring two attempts over 11 min. The procedure was uneventful, with spontaneous breathing maintained throughout, heart rate between 80–95 bpm, SpO2 97%–100% and EtCO2 22-45 mmHg.

We monitored the patient while asleep, and her vital parameters remained normal.

Fifty minutes after the procedure, the girl awoke agitated and crying, complaining of breathing difficulty and chest pain. The physical examination was unremarkable, and the staff calmed her.

After 10 min, she appeared to fall into a deep sleep again, presenting a prolonged apnea with significant desaturation (SpO2 55%). Despite verbal and tactile stimulation, she was difficult to arouse; a brief period of valve mask ventilation was performed, which resulted in prompt arousal and eventual normalization of the breathing pattern. At this point, she became fully responsive to verbal commands.

Over the next 5 h, the girl continued to show a respiratory rate at the lower limits while awake (average 12–14 breaths per minute), experiencing three more episodes with a worsening to bradypnea and apnoea when inattentive, more drowsy or with eyes closed. In these occasions, the slowing of the respiratory rate was always followed by quick desaturation, with one value as low as 80% in the second episode. On this second occasion, verbal stimulation was not enough, and a bag valve mask ventilation was restarted, but it promptly resolved with the physical stimulation. We settled the other two episodes with verbal stimulation and the awakening.

After the episodes we performed a blood gas analysis and a glucose level, to assess the patient’s respiratory and metabolic function. Given the history of vomiting shortly after the induction of sedation, a chest X-ray was also performed to rule out aspiration of gastric contents. Finally, an MRI of the brain was conducted to assess for possible signs of central nervous system involvement, such as ischemia or Posterior Reversible Encephalopathy Syndrome (PRES), despite the absence of typical signs or symptoms. PRES could be characterized by seizures at onset and could have been related to the use of methotrexate or other drugs, which were part of the patient’s standard treatment. All tests were within normal limits.

**FIGURE 1:**
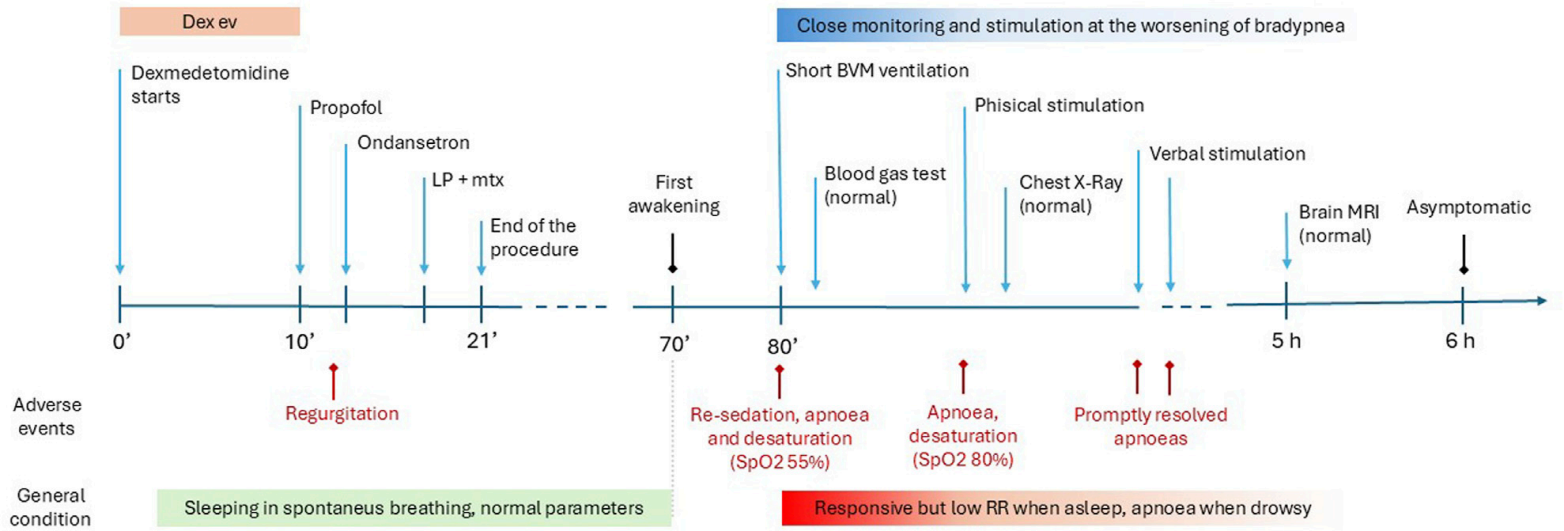
Timeline. This figure shows the events from the case report in chronological order. Medical interventions are indicated at the top of the line; LP + mtx (lumbar puncture medicated with methotrexate); BVM ventilation (bag-valve-mask ventilation); Brain RMI (Brain Magnetic Resonance Imaging). Adverse events (in red) and general conditions are represented in the lower part; RR (respiratory rate).

The girl was fully awake 5 h after sedation, and no further episodes occurred. She prudentially remained under close monitoring until the following morning and was discharged without complications A timeline showcasing the relationship between patient’s medication and reaction time is reported in Figure 1.

Patient’s previous history was remarkable for being delayed methotrexate elimination. In fact, during high-dose methotrexate administrations, the girl exhibited prolonged elimination clearance with blood level reaching <0.25 mmol in 66–78 h. However, genetic investigations had excluded mutations affecting specific metabolizing enzymes of the drug. Her previous sedation history was unremarkable, with no adverse events and normal awakening times after multiple sedations with midazolam and propofol, and a single previous sedation with dexmedetomidine and propofol, albeit with a lower total propofol dose (Table 1).

**TABLE 1:** Summary of patient’s procedures, with dosages of sedative drugs. The bold data correspond to the recurrent sedation and apnoea episode.

Procedure Date	Procedure type	Midazolam	Propofol	Dexmetomedine	Ketamine
28 June 2023	Bone marrow aspirate + central venous catether positioning	2 mg	300 mg		100 mg
10 July 2023	Bone marrow aspirate + lumbar puncture	2 mg	450 mg		
14 August 2023	Lumbar puncture	1 mg	340 mg		
28 August 2023	Lumbar puncture	5 mg	240 mg		
10 October 2023	Bone marrow aspirate	5 mg	260 mg		
8 November 2023	Lumbar puncture	5 mg	320 mg		
23 November 2023	Lumbar puncture	5 mg	200 mg		
7 December 2023	Lumbar puncture		200 mg		
28 December 2023	Lumbar puncture	4 mg	330 mg		
18 January 2024	Bone marrow aspirate		200 mg	54 mcg	
5 Marc^h^ 2024	Lumbar puncture	5 mg	165 mg		
12 Marc^h^ 2024	Lumbar puncture		**290 mg**	**55 mcg**	

Pharmacological studies were conducted to investigate the molecular mechanism underlying late-onset sedation and apnea. Plasma concentrations of dexmedetomidine and propofol were measured in blood samples collected 20 h after the adverse reaction, resulting very low (dexmedetomidine: 0.03 ng/mL and propofol: 0 ng/mL). Genotyping was performed on approximately 1,000,000 single nucleotide polymorphisms (SNPs) across the entire genome using the Illumina Infinium CytoSNP-850K v1.4 BeadChip. Written informed consent was obtained from the patient and her parents. The selection of candidate genes and variants involved in dexmedetomidine and propofol pharmacokinetics and pharmacodynamics was accomplished using the Pharmacogenomics Knowledge Base (PharmGKB). In particular (Table 2), we identified 26 genes with variants associated with drug responses: 12 genes for dexmedetomidine, 12 genes for propofol, and 2 genes linked to both drugs. The platform enabled the genotyping of 1,488 variants across these genes. The patient tested homozygous for one relatively rare variant (minor allele frequency below 10%) in the European population (dbSNP). Specifically, the variant (rs16851751) is in the 3′-UTR regulatory region of the *SCN9A* gene, encoding for a sodium channel. Sanger sequencing confirmed the variant. Notably, the variant was absent in 43 patients undergoing the same sedation protocol without adverse events.

**TABLE 2:** Candidate genes evaluated, number of variants in each gene and drug associated.

Gene name	Number of variants genotyped by array illumina infinium CytoSNP-850K v1.4 BeadChip	Drug related according to PharmGKB variant annotation
ABCB1	151	propofol
ABCC9	95	dexmedetomidine
ABCG2	86	dexmedetomidine
ADRA2A	2	dexmedetomidine
ADRB2	10	dexmedetomidine, propofol
ATP8B3	16	propofol
CACNB2	205	dexmedetomidine
CYP1A2	11	dexmedetomidine
CYP2A6	7	dexmedetomidine
CYP2B6	25	propofol
CYP2C9	71	propofol
CYP2D6	6	dexmedetomidine
DRD2	24	propofol
GABRA1	9	propofol
GABRA2	17	dexmedetomidine, propofol
HTR2A	45	propofol
KCNA3	1	dexmedetomidine
KCNE1	31	propofol
KCNE2	5	propofol
KCNMA1	284	dexmedetomidine
KCNMB1	11	dexmedetomidine
PRKCB	117	dexmedetomidine
RYR1	46	propofol
SCN9A	58	propofol
UGT1A9	149	propofol
WBP2NL	6	dexmedetomidine

## Discussion

This study is the first case report of post-sedation apnoea episodes following short procedural sedation with dexmedetomidine and propofol. Remarkably, the patient could be aroused with significant verbal and physical stimulation, including a brief ventilation cycle, during the first two episodes, with spontaneous recovery of breathing and saturation. However, she fell asleep again and presented two further additional apnoeic episodes over 3 h. Of note, her previous sedation history was unremarkable, even if the association with propofol and dexmedetomidine had been previously used only once, and with a lower propofol dose.

Full arousal after dexmedetomidine sedation may be pretty long (in the range of hours), and recurrent sleep phases after an initial awakening are typical in children. However, these sleep phases are typically not associated with apnoea or severe desaturation (Lee, 2019), as observed in this case. Similarly, propofol, when used as monotherapy, has not been associated with re-sedation or desaturation long after a procedure once complete awakening has occurred (Kim et al., 2019).

Dexmedetomidine is a highly selective alpha2-agonist with anxiolytic and sedative properties. The drug induces natural sleep comparable to stages 2–3 of non-REM sleep by stimulating alpha adrenergic receptors in the locus coeruleus (Lee, 2019; Gertler et al., 2001). Unlike most sedatives, such as opioids and benzodiazepines, dexmedetomidine provides arousable and interactive sedation without significant respiratory depression. Literature reports a certain analgesic effect and therefore dexmedetomidine is considered a non-opioid adjuvant analgesic drug in perioperative acute pain management (Tang and Xia, 2017). However, in clinical practice, its use as a single drug is not usually considered for significantly painful procedures.

The most common side effects include bradycardia and hypotension, although these rarely require medical support. When combined with other sedatives, dexmedetomidine enhances anaesthetic and sedative effects, increasing the risk of cardiorespiratory depression (Gertler et al., 2001).

Additionally, dexmedetomidine pharmacokinetics exhibit significant inter-individual variability influenced by body size, ethnicity, hepatic function, plasma albumin levels and cardiac output (Zhang et al., 2023).

Propofol, on the other hand, can cause respiratory depression at higher doses, especially when rapidly infused. However, due to its short half-life, it has not been associated with delayed re-sedation or apnea (Chidambaran et al., 2015).

Concerning dexmedetomidine, only one case (Ho et al., 2005), of central apnea following general anesthesia has been reported in the literature, though several differences distinguish it from ours. Firstly, in that report, an adult patient underwent general anesthesia with tracheal intubation and induction using two agents with respiratory depressant effect (fentanyl and propofol). The continuous infusion of dexmedetomidine was started 50 min after induction as a maintenance agent for pain management after surgery; the cumulative dose of dexmedetomidine was the same as in our case (1 mcg/kg). A single apneic episode occurred shortly after admission to the post-anesthetic-care unit and removal of the tracheal tube; during this episode, dexmedetomidine infusion was promptly discontinued. Subsequently, the patient fully awoke within 5 min, and no further episodes occurred.

In literature, there is another case of altered breathing patterns in a patient who was administered dexmedetomidine to sedate a state of delirium (Balavenkataraman et al., 2022). The continuous infusion initially involved a dose of 1.5 mcg/kg/h. After about 90 min, the patient exhibited an alternating apnea and hyperpnea pattern of breathing with a crescendo-decrescendo pattern characteristic of central sleep apnea. The breathing pattern normalized rapidly after reducing the infusion rate to 0.5 mcg/kg/h, and there was no further recurrence of central sleep apnea.

In contrast to both cases, our patient experienced a prolonged awakening period, almost 4 h, characterized by multiple episodes of recurrent drowsiness with hypopnea. Dexmedetomidine infusion was stopped before induction with propofol, and no additional drugs were administered following the procedure.

Considering the short plasma elimination half-life of dexmedetomidine (approximately 2.5 h) and its target plasma concentration (0.2–0.6 ng/mL) (Weerink et al., 2017), the low concentrations measured 24 h after the event exclude reduced metabolism and drug elimination. A peculiar pharmacogenetic profile and the association of propofol and dexmedetomidine could have contributed to these events.

Our patient was homozygous for a relatively rare variant (minor allele frequency ≤10%) in the European population (dbSNP). Interestingly, an analysis of 43 samples from patients who underwent the same sedation but experienced a normal course did not detect this variant, even in the heterozygous state. Specifically, the variant rs16851751 in the *SCN9A* gene, encoding a voltage-dependent sodium channel subunit, is of particular interest. Variants in this gene have been associated with deep anesthesia induced by propofol (Zhong et al., 2017). Dexmedetomidine has been reported to inhibit the function of this channel, by inhibitory G proteins activated by its target, the α2-adrenoceptor (Im et al., 2018; Ding et al., 2023). Interestingly, SCN9A inhibitors have analgesic properties, and non-specific inhibition of these channels has been related to respiratory cessation (Klein et al., 2022). This relatively rare variant contributed to the patient’s abnormal response. The variant is located in the 3′-UTR regulatory region of *SCN9A*, and it may affect the stability of the mRNA, making the patient more susceptible to the effects of propofol and dexmedetomidine, even if it is not described as an eQTL for this or other genes (GTEX portal accessed on 10/29/2024). A puzzling issue concerns the fact that the patient had been previously sedated once with the same drug regimen (propofol and dexmedetomidine) without adverse effects. However, it is well reported in the literature that drug-related adverse events caused by this kind of variant do not necessarily take place at every drug administration, with a variability possibly related to other factors such as genetic background, environmental influences, or pharmacokinetic variability (Paugh et al., 2010).

Our study’s limitations include the absence of drug concentration measurements during therapy, which could have provided valuable insights into the appropriate exposure levels for both medications. Additionally, although our genetic analysis was comprehensive, employing more detailed approaches such as exome or whole-genome sequencing might have identified rare variants that influence patient responses.

We believe that this case deserves to be shared to make physicians aware of the risk of delayed re-sedation and apnoea after dexmedetomidine and propofol sedation in patients with a unique pharmacogenetic phenotype. This study contributes to current understanding of adverse events during anesthesia; if validated by further evidence, pharmacogenetic testing may be included in guidelines for anesthesia in patients with rare genetic variants.

## Data Availability

The original contributions presented in the study are publicly available. This data can be found here: ClinVar, accession number SCV005902348.

## References

[B1] Balavenkataraman A. Gopal N. Moss J. E. Arunthari V. Colaco B. (2022). Dexmedetomidine as a potential cause of central sleep apnea. Am. J. Respir. Crit. Care Med. 205, A4596.

[B2] Banerjee P. Rossi M. G. Anghelescu D. L. Liu W. Breazeale A. M. Reddick W. E. (2019). Association between anesthesia exposure and neurocognitive and neuroimaging outcomes in long-term survivors of childhood acute lymphoblastic leukemia. JAMA Oncol. 5 (10), 1456–1463. 10.1001/jamaoncol.2019.1094 31219514 PMC6587141

[B3] Chidambaran V. Costandi A. D'Mello A. (2015). Propofol: a review of its role in pediatric anesthesia and sedation. CNS Drugs 29 (7), 543–563. 10.1007/s40263-015-0259-6 26290263 PMC4554966

[B4] Ding Y. Liu A. Wang Y. Zhao S. Huang S. Zhu H. (2023). Genetic polymorphisms are associated with individual susceptibility to dexmedetomidine. Front. Genet. 14, 1187415. 10.3389/fgene.2023.1187415 37693312 PMC10483403

[B5] Gertler R. Brown H. C. Mitchell D. H. Silvius E. N. (2001). Dexmedetomidine: a novel sedative-analgesic agent. Proc. Bayl Univ. Med. Cent. 14 (1), 13–21. 10.1080/08998280.2001.11927725 16369581 PMC1291306

[B6] Ho A. M. Chen S. Karmakar M. K. (2005). Central apnoea after balanced general anaesthesia that included dexmedetomidine. Br. J. Anaesth. 95 (6), 773–775. 10.1093/bja/aei263 16243902

[B7] Im S. T. Jo Y. Y. Han G. Jo H. J. Kim Y. H. Park C. K. (2018). Dexmedetomidine inhibits voltage-gated sodium channels via α2-adrenoceptors in trigeminal ganglion neurons. Mediat. Inflamm. 2018, 1782719. 10.1155/2018/1782719 PMC6139198 30245586

[B8] Kim S. Hahn S. Jang M. J. Choi Y. Hong H. Lee J. H. (2019). Evaluation of the safety of using propofol for paediatric procedural sedation: a systematic review and meta-analysis. Sci. Rep. 9 (1), 12245. 10.1038/s41598-019-48724-x 31439875 PMC6706375

[B9] Klein R. M. Layton M. E. Regan H. Regan C. P. Li Y. Filzen T. (2022). Association of respiratory failure with inhibition of NaV1.6 in the phrenic nerve. Channels (Austin) 16 (1), 230–243. 10.1080/19336950.2022.2122309 36239534 PMC9578445

[B10] Lee S. (2019). Dexmedetomidine: present and future directions. Korean J. Anesthesiol. 72 (4), 323–330. 10.4097/kja.19259 31220910 PMC6676029

[B11] Paugh S. W. Stocco G. Evans W. E. (2010). Pharmacogenomics in pediatric leukemia. Curr. Opin. Pediatr. 22 (6), 703–710. 10.1097/MOP.0b013e32833fde85 20861736 PMC3612020

[B12] Tang C. Xia Z. (2017). Dexmedetomidine in perioperative acute pain management: a non-opioid adjuvant analgesic. J. Pain Res. 10, 1899–1904. 10.2147/JPR.S139387 28860845 PMC5565238

[B13] Weerink M. A. S. Struys M. M. R. F. Hannivoort L. N. Barends C. R. M. Absalom A. R. Colin P. (2017). Clinical pharmacokinetics and pharmacodynamics of dexmedetomidine. Clin. Pharmacokinet. 56 (8), 893–913. 10.1007/s40262-017-0507-7 28105598 PMC5511603

[B14] Zhang J. Yin J. Li Y. Zhang Y. Bai Y. Yang H. (2023). Effect of dexmedetomidine on preventing perioperative respiratory adverse events in children: a systematic review and meta-analysis of randomized controlled trials. Exp. Ther. Med. 25 (6), 286. 10.3892/etm.2023.11985 37206556 PMC10189613

[B15] Zhong Q. Chen X. Zhao Y. Liu R. Yao S. (2017). Association of polymorphisms in pharmacogenetic candidate genes with propofol susceptibility. Sci. Rep. 7 (1), 3343. 10.1038/s41598-017-03229-3 28611364 PMC5469860

